# Visual statistical learning in children and young adults: how implicit?

**DOI:** 10.3389/fpsyg.2014.01541

**Published:** 2015-01-08

**Authors:** Julie Bertels, Emeline Boursain, Arnaud Destrebecqz, Vinciane Gaillard

**Affiliations:** ^1^Faculty of Psychology and Educational Sciences, Center for Research in Cognition and Neurosciences, Université Libre de Bruxelles, Brussels, Belgium; ^2^Fonds de la Recherche Scientifique – Fonds National de la Recherche Scientifique, Brussels, Belgium

**Keywords:** visual statistical learning, children, consciousness, confidence judgments, implicit and explicit knowledge

## Abstract

Visual statistical learning (VSL) is the ability to extract the joint and conditional probabilities of shapes co-occurring during passive viewing of complex visual configurations. Evidence indicates that even infants are sensitive to these regularities (e.g., Kirkham et al., 2002). However, there is continuing debate as to whether VSL is accompanied by conscious awareness of the statistical regularities between sequence elements. Bertels et al. (2012) addressed this question in young adults. Here, we adapted their paradigm to investigate VSL and conscious awareness in children. Using the same version of the paradigm, we also tested young adults so as to directly compare results from both age groups. Fifth graders and undergraduates were exposed to a stream of visual shapes arranged in triplets. Learning of these sequences was then assessed using both direct and indirect measures. In order to assess the extent to which learning occurred explicitly, we also measured confidence through subjective measures in the direct task (i.e., binary confidence judgments). Results revealed that both children and young adults learned the statistical regularities between shapes. In both age groups, participants who performed above chance in the completion task had conscious access to their knowledge. Nevertheless, although adults performed above chance even when they claimed to guess, there was no evidence of implicit knowledge in children. These results suggest that the role of implicit and explicit influences in VSL may follow a developmental trajectory.

## INTRODUCTION

Statistical learning (SL) refers to the learning mechanisms that subtend sensitivity to the statistical regularities present in the environment. This process is essential when facing a complex environment. In particular, learning of transitional probabilities is crucial to predict upcoming events on the basis of previous ones. Congruently, many authors have documented such an ability to extract statistical regularities from auditory and visual inputs in adults, children and infants (for a review of SL across development, see Krogh et al., 2013).

Statistical learning is usually thought of as a form of implicit learning, which is a fundamental and ubiquitous process in cognition (Perruchet and Pacton, 2006). As a matter of fact, the occurrence of SL has been observed in infants (Saffran et al., 1996; Kirkham et al., 2002; Bulf et al., 2011) as well as in non-human primates (Goujon and Fagot, 2013; Rakoczy et al., 2014). Moreover, SL occurs incidentally, that is even when participants are not informed about the existence of regularities in the material. It also occurs outside the focus of attention, that is, even when participants are engaged in an unrelated concurrent task (e.g., Saffran et al., 1997; but see Turk-Browne et al., 2005). Remarkably, SL is observed even though most participants do not report any conscious knowledge of the regularities (e.g., Kim et al., 2009; Arciuli and Simpson, 2011, 2012).

Therefore, the knowledge acquired through SL has generally been taken to be implicit, a perspective that is tacitly assumed by most authors in this domain, who generally tend to consider that any SL process always takes place outside awareness. However, this assumption has seldom been tested directly.

To the best of our knowledge, Kim et al. (2009) were the first to tackle this issue. These authors investigated whether the statistical regularities between geometrical shapes can be learned outside awareness. Based on a dissociation between direct and indirect measures of learning (i.e., in which task instructions respectively do or do not explicitly refer to the to-be-learned dimension), they concluded that participants learned the sequences implicitly. However, in a replication of their study using more sensitive and subjective measures of the acquired knowledge, Bertels et al. (2012) demonstrated that participants’ performance was not exclusively based on implicit knowledge. On the contrary, visual statistical learning (VSL) was mostly based on explicit knowledge acquisition (see also Bertels et al., 2013).

In their studies, following Kim et al. (2009), Bertels et al. (2012, 2013) first exposed participants to a stream of stimuli made up of the repeated presentation of 12 shapes, each of them being part of a triplet, namely a sequence of three visual shapes presented successively in a fixed order. Crucially, triplets could not be segmented on the basis of any spatial or temporal cue, as each shape was centrally presented and as the inter-stimulus interval was held constant within and across triplets. The transitional probabilities between shapes are therefore stronger within a triplet (*P* = 1.0) than between any two triplets (*P* = 0.33). After this exposure phase, participants were faced with a rapid serial visual presentation (RSVP) test, in which they had to detect as fast as possible a target shape in a continuous stream made by the random succession of the triplets. The rationale was that if participants learned the statistical regularities of the triplets during the exposure phase, reaction times (RTs) should be faster to the second and third, predictable elements of each triplet than to the first, unpredictable element. In a subsequent completion task, participants had to choose on each trial the missing shape in a series of to-be-completed triplets. They had to choose between four alternatives and were also asked to evaluate on a binary scale whether they remembered or guessed which one was the missing shape. Here, we use a similar paradigm in order to investigate the implicit vs. explicit nature of VSL in children.

Learning of statistical contingencies, be they spatial or temporal, has been studied in typically developing children (e.g., Saffran et al., 1997; Arciuli and Simpson, 2011, 2012; Couperus et al., 2011) as well as in specific populations such as children with high functioning autism (Mayo and Eigsti, 2012) and children with specific language impairment (Evans et al., 2009). Most studies reported effective learning, supporting that SL is an early maturing ability. Nevertheless, research in children suffers from the same limitation as the adult literature. The extent to which SL can actually take place outside participants’ awareness has indeed seldom been addressed directly.

In the field of implicit learning however, the implicit vs. explicit nature of the knowledge acquired by children has been addressed. For instance, using the sequence learning paradigm, Thomas and Nelson (2001) distinguished between participants who had gained explicit knowledge of the sequence or not, and observed that the explicit group showed larger learning effects than the implicit group. Recently, using the same paradigm, Weiermann and Meier (2012) even reported that children only learned the sequences when they acquired substantial explicit knowledge. These results highlight the importance of considering the nature of the acquired knowledge in developmental studies of SL, as conscious knowledge may in fact exert a substantial effect on performance.

In the present study, we adapted Bertels et al.’s (2012) paradigm to children, that is, we slowed down the pace of the tasks and made them as engaging as possible. Our procedure departs from previous studies on VSL in children (e.g., Arciuli and Simpson, 2011, 2012) for two main reasons. First, we used an indirect measure of learning (i.e., the RSVP detection task) in addition to the direct measure (here, a completion task). As participants are required to respond on the basis of their explicit knowledge in the direct but not in the indirect task, if the indirect task detects some knowledge that is left undetected by the direct task, this knowledge can be considered as unconscious (Reingold and Merikle, 1988). Second, we used subjective measures of performance (i.e., confidence judgments), in addition to objective measures. To the best of our knowledge, this is the first study that uses confidence judgments with children in a SL task (but see, e.g., Berch and Evans, 1973, for the use of confidence judgments in an explicit memory task).

The combined use of confidence judgments and objective measures of performance will allow us to investigate the explicit vs. implicit nature of the acquired knowledge. If children learn explicitly, knowledge should not only be above the *objective threshold* (the chance level in the completion task), but also above the *subjective threshold* (Cheesman and Merikle, 1984): they should be more confident in correct than in incorrect completion trials, based on the notion that they should know when they are using their statistical knowledge. This relation between confidence and accuracy indicates conscious knowledge by the *zero-correlation criterion* (Dienes et al., 1995). If learning takes place implicitly, performance should be above the objective but below the subjective threshold of consciousness as performance and confidence should not be related to each other. Rather, performance should be above chance even when participants claim to guess in the completion task, which indicates unconscious knowledge by the *guessing criterion* (Dienes et al., 1995).

We tested a sample of four- and fifth-graders as Koriat and Ackerman (2010) demonstrated that second- and third-graders are significantly less able than older children to monitor the correctness of their answers. We also recruited a group of young adults and used the exact same methodology. This comparative approach allowed us to assess the effect of age (1) in learning statistical regularities from sequentially presented visual information (as assessed through RTs in the RSVP task), (2) in using the acquired knowledge in a subsequent direct task (as assessed by completion performance), (3) in expressing confidence in the rightness of one’s own responses (as assessed by confidence ratings), and, crucially, (4) in consciously accessing the acquired knowledge (as assessed by the correlation between completion scores and confidence ratings).

Based on previous studies showing effective SL in children and adults (e.g., Arciuli and Simpson, 2011, 2012; Bertels et al., 2012, 2013), we predict successful learning in both groups of participants, as reflected by (1) faster detection of shapes appearing in predictable locations in the RSVP task (even though we expect children to respond slower than young adults overall), and (2) above-chance performance in the completion task.

Previous studies using direct measures of learning have demonstrated that SL follows a developmental trajectory whereby learning improves with age (e.g., Vaidya et al., 2007; Arciuli and Simpson, 2011, 2012; but see Saffran et al., 1997). Age effects have also been reported in the broader field of implicit learning, at least when participants were explicitly instructed to learn a sequence (Karatekin et al., 2007) or when explicit instructions were used at test while learning was incidental (Witt et al., 2013). We therefore expect to observe better performance in adults than in children in the direct completion task.

Analysis of the subjective confidence judgments will be informative concerning the explicit or implicit nature of SL. Regarding young adults, we expect to replicate results from Bertels et al. (2012, 2013) namely that VSL is based on a mixture of implicit and explicit knowledge. What about the effect of age on the implicit vs. explicit nature of learning? Even though consciousness has become the topic of numerous studies during the past 20 years, very few of them addressed the issue of the development of conscious awareness. Most theoretical models consider that, during development, some form of improvement takes place regarding the cognitive abilities generally associated with conscious processing in adults, such as verbal access, explicit memory, and cognitive control (Perner and Dienes, 2003; Zelazo, 2004; Cleeremans, 2006). Recently, Daltrozzo and Conway (2014) developed a model of statistical-sequential learning across the life span, according to which the “basic” and “expert” systems composing SL would differ regarding their developmental trajectories. The “basic” system would mostly depend on automatic and implicit mechanisms. Available since birth, its contribution would decline until adulthood and then grow again in old age. Conversely, the “expert” system would rely on top-down explicit mechanisms. It would gradually develop from the early childhood into adulthood, and then decline in the elderly. The basic and the expert systems would thus coexist early in life, but their contribution to SL would develop inversely.

As a consequence, we do not expect learning to be exclusively implicit in the children group. Rather, we expect that, as adults, their performance would rely on the acquisition of both implicit and explicit knowledge. Nevertheless, following Daltrozzo and Conway’s (2014) model, we expect a qualitative dissociation regarding the respective influences of implicit and explicit learning in both age groups. Specifically, children would rely more on implicit and less on explicit knowledge than adults. We thus expect higher performance in children than in adults when they correctly guessed the missing shape in the completion task. We also expected a larger Chan difference in adults than in children. In other words, we expect the difference in confidence between correct and incorrect responses to be larger in adults than in children.

## MATERIALS AND METHODS

### PARTICIPANTS

One hundred and seven individuals took part in the study. All reported (corrected-to-) normal vision. There were two groups. The first group consisted of 54 fourth and fifth graders (24 girls), typically developing (i.e., from general education and with no reported disabilities), ranging from 9 years and 7 months old to 12 years and 3 months old (*M* = 10 years and 9 months old). They were recruited from three primary schools (one in Belgium, two in France, all French-speaking) and volunteered for the study after one of the parents had given their informed consent. The data from six participants (three girls) were discarded from the analyses: two because the experiment had to be interrupted before the end, two because they did not pay enough attention to the stream during Exposure (namely, their average detection rate of cartoon characters in the cover task was more than two standard deviations below the mean performance), and two because their average RT on the RSVP task was more than two standard deviations above the overall average RT.

The second group consisted of 53 students of the Université Libre de Bruxelles (45 women), ranging from 17 to 24 years old (*M* = 19 years and 9 months old). They were tested at the Center of Research in Cognition and Neurosciences and received course credits for their participation. The data from three participants (three women) were discarded from the analyses: two because their average RT on the RSVP task was more than two standard deviations above the overall average performance, and one because their mean error rate was above the same criterion.

This study was approved by the Ethics Committee of the Psychological and Educational Sciences Faculty of the Université Libre de Bruxelles and conforms to the relevant regulatory standards.

### STIMULI

**FIGURE 1:**
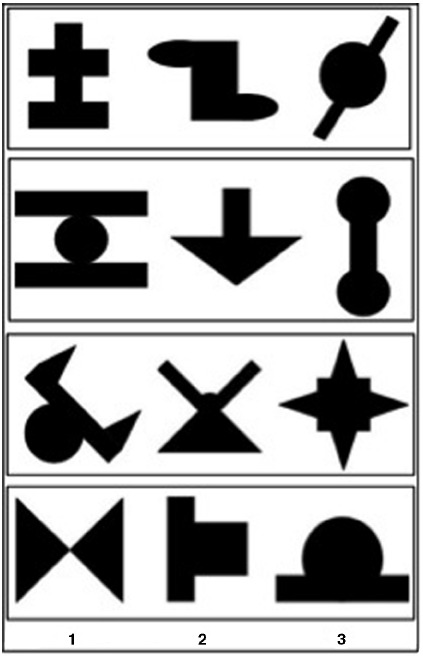
**Groups of three shapes constituting each of the four triplets, by order of presentation (1, 2, 3)**.

The visual stimuli consisted of 12 black shapes presented on a white background, adapted from Fiser and Aslin (2001). Each stimulus was about 3 cm wide and 3 cm high. Stimuli constituted four “triplets,” namely four sequences of three stimuli presented in a fixed order (Figure 1). Similarly to previous studies (Arciuli and Simpson, 2011, 2012) and as no effect of stimulus make-up was previously found (Bertels et al., 2012), we only used one arrangement of four triplets in the present study.

### APPARATUS

Stimulus presentation, timing, and data collection were controlled using the Psyscope USB button box and Psyscope X software (Cohen et al., 1993) running on a MacBook 2.26 GHz Intel Core 2 Duo.

### PROCEDURE

The exposure phase was followed by a surprise test phase consisting of a RSVP detection task (indirect measure of learning) and a four alternatives forced choice (4AFC) completion task (direct measure).

#### Exposure

Exposure consisted of 1260 trials divided in four blocks, each of them consisting of 25 repetitions of each of the 12 shapes constituting the triplets plus 15 trials consisting in the presentation of a cartoon character (the Marsupilami, Homer Simpson, Bill, or Titeuf, depending on the block). Stimuli were presented one at a time, for 250 ms, with a 250 ms ISI (for similar presentation rates, see Bertels et al., 2013), resulting in an exposure phase of about 10 min. Each of the 12 shapes was presented in the fixed order defined by the triplet it was part of. Participants were not told about the presence of regularities in the sequence. Triplets were pseudo-randomly presented: a given triplet was never presented twice in a row. The presentation of the shapes was randomly interspersed with the presentation of the cartoon character. As a cover task, participants were asked to detect the cartoon character by pressing the right key. This procedure was used to ensure that participants paid attention to the stimuli presented in the exposure phase without explicitly drawing their attention to the sequence of shapes. These data were not considered in the analyses but were used to discard participants who did not detect enough of them (see above).

#### RSVP task

The first test consisted in a RSVP paradigm in which participants had to detect a target within a stream of stimuli. On each trial, the presentation of the target (1 of the 12 shapes presented during Exposure) was followed by the presentation of the four triplets, one shape at time, at the same rate as during Exposure. Participants were asked to press the right key as soon as they saw the target. The RSVP stream was then interrupted, and the next target was presented. The RSVP task began with two practice trials. Then, each target shape was presented four times, in the second or third triplet in the RSVP stream, resulting in 48 test trials. Trials were randomly presented across participants.

#### Completion task

The second test consisted in a 4AFC task in which participants were presented with a triplet in which one shape was missing. They were informed that these triplets were presented before. On each trial, the triplet was first presented twice, one shape at a time at the same rate as during Exposure, with a question mark in place of the missing shape. Then, the three shapes (including the question mark) were displayed side by side on the top of the screen, in the order defined by the triplet. Participants had to pick one shape among four presented underneath to complete the triplet. These shapes were part of the triplets presented before, and their position corresponded to the position of the missing shape in the to-be-completed triplet. Participants responded by pressing one of four keys (in case of children, the experimenter pressed the key corresponding to the child’s answer). No time constraint was imposed.

The completion task began with two practice trials for which participants received no feedback. Then, each triplet was presented six times, resulting in 24 test trials. The same missing shape in the first, second or third location was thus presented twice, with a different presentation order of the four possible shapes. The sequence of completion trials was randomly presented across participants.

After each trial, participants expressed a binary confidence judgment regarding their completion response. They had to indicate whether they had guessed (i.e., they had no idea whatsoever concerning the correct response, they had answered at random) or remembered (i.e., they felt that their response was based on some recall of the learning material, even if they were not sure at all) by pressing one of two keys (for similar labels, see Dienes and Seth, 2010; Bertels et al., 2012, 2013). Again, the experimenter pressed the key corresponding to the child’s answer.

## RESULTS

### DID PARTICIPANTS LEARN THE STATISTICAL REGULARITIES?

In the RSVP task, misses and erroneous detections consisted of 13.108% of the trials in the group of children, and 6.208% of the trials in the group of young adults. A one-way analysis of variance (ANOVA) showed that the percentage of errors was significantly larger in children than in young adults, *F*(1,97) = 39.132, *p* < 0.001. Subsequent analyses were performed on correct RTs only.

**FIGURE 2:**
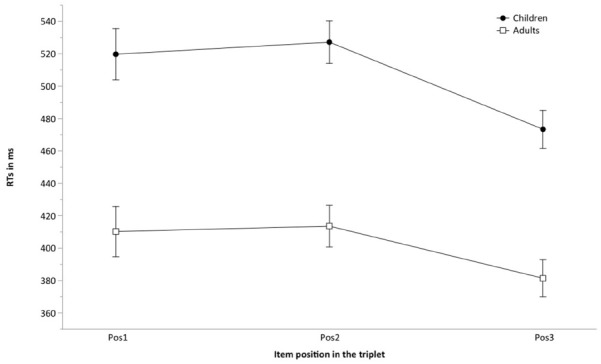
**Mean detection latencies for the three item positions (Pos1, Pos2, Pos3) in the RSVP task, by group of participants.** Error bars represent 95% of confidence intervals around the means.

A mixed ANOVA was applied on response latencies, with Group (two levels: children, adults) as a between-subjects factor and Position (three levels: 1, 2, 3) as a within-subject factor. This analysis revealed a significant effect of Group, *F*(1,96) = 182.837, *p* < 0.001, ${\text{η}}_{\text{p}}^{\text{2}}$ = 0.656, indicating that young adults responded faster than children (402 vs. 507 ms). Crucially, the effect of Position was also significant, *F*(2,192) = 44.704, *p* < 0.001, ${\text{η}}_{\text{p}}^{\text{2}}$ = 0.318. Bonferroni adjusted comparisons revealed that RTs in Position 3 (427 ms) were significantly faster than in Positions 1 and 2 (465 and 470 ms), both *p* < 0.001, which failed to differ from each other, *F* < 1. The interaction between Group and Position was not significant, *F*(2,192) = 2.664, *p* = 0.072, ${\text{η}}_{\text{p}}^{\text{2}}$ = 0.027^1^ (see Figure 2). These results indicate that, on average, participants had learned the triplets.

### DID PARTICIPANTS EXPLOIT THEIR STATISTICAL KNOWLEDGE IN THE COMPLETION TASK?

Completion performance across age groups was 29.762% (SD = 14.67%) and differed significantly from chance-level (25%), *t*(97) = 3.211, *p* = 0.002. This result indicates that, overall, participants used their sequence knowledge in the completion task.

A non-parametric Mann–Whitney test revealed, however, that the proportion of correctly completed trials did not significantly differ between children (27.431%, SD = 11.936%) and young adults (32.01%, SD = 16.713%; *U* = 1084.5, *p* = 0.408)^2^. In line with this result, the proportion of participants who performed at chance (i.e., obtaining only 25% or less of correct responses, *n* = 45) did not differ between groups (*n* = 23 in the children group and *n* = 22 in the young adults group, *χ*^2^(1, *n* = 98) = 0.151, *p* = 0.697).

### WERE PARTICIPANTS CONFIDENT IN THEIR COMPLETION PERFORMANCE?

Overall, participants judged that they remembered which was the missing shape in 54.167% of the cases (SD = 26.508%) and that they guessed in the remaining 45.833%. A one-way ANOVA revealed that confidence (i.e., the percentage of “remember” responses) significantly differs between age groups, *F*(1,97) = 9.489, *p* = 0.003. While children judged that they remembered in 62.24% of the cases (SD = 19.814%), young adults’ confidence dropped to 46.417% (SD = 29.822%). Five participants (one child, four adults) claimed to guess on every trial and therefore reported no “remember” responses. Two participants (one child, one adult) claimed to remember on every trial. It thus appears that children have a liberal metacognitive bias, namely a “tendency to give high confidence ratings, all else being equal” (Fleming and Lau, 2014, p. 5).

### DID PARTICIPANTS HAVE CONSCIOUS ACCESS TO THEIR STATISTICAL KNOWLEDGE?

We limited this analysis to participants who performed above chance in the completion task (*n* = 53), that is who acquired knowledge of the statistical contingencies according to the direct measure of learning (for a similar procedure, see Bertels et al., 2012, 2013). We investigated whether their knowledge was above the subjective criterion of consciousness, namely whether they had metaknowledge about their statistical knowledge. To this aim, we used two indicators often used in implicit learning studies (see Gaillard et al., 2014, for a review): the zero-correlation criterion (Chan, 1992) and the guessing criterion (Dienes et al., 1995).

The zero-correlation criterion is met when there is no relationship between confidence and performance. In other words, if high and low confidence ratings are randomly assigned to correct and incorrect discriminations. Conversely, if performance is based on conscious knowledge, participants should be more confident in their correct responses than in their errors (Chan, 1992). According to the guessing criterion, knowledge is below the subjective threshold of consciousness when performance is above chance while participants claim to guess.

Completion performance reached 36% of correct responses (SD = 9.151%) in children (*n* = 25) and 42.56% (SD = 15.06%) in young adults (*n* = 28). Children who performed above chance reported to remember the missing shape in 63.5% of the cases (SD = 21.425%), while young adults who performed above chance reported to remember it in 53.72% of the cases (SD = 29.427%).

**FIGURE 3:**
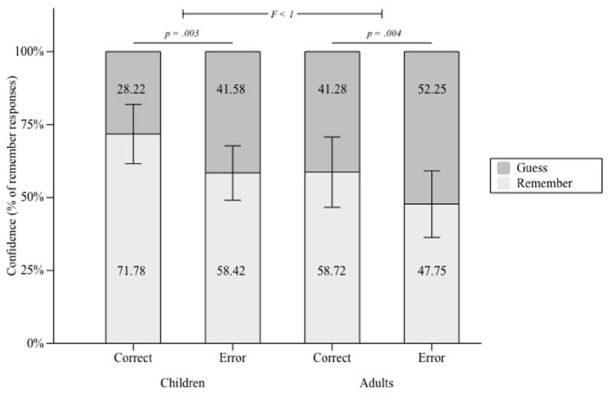
**Proportion of Remember and Guess responses in participants who performed above chance level in the completion task, represented separately for correct and incorrect completions, in children and adults.** Error bars represent 95% of confidence intervals around the means.

We observed that participants who performed above chance in the completion task made significantly more “remember” judgments when they were correct than incorrect (Chan difference = 12.097, SD = 19.131), *t*(52) = 4.603, *p* < 0.001, were they children (Chan difference = 13.355, SD = 20.263) or young adults (Chan difference = 10.973, SD = 18.361), *t*(24) = 3.295, *p* = 0.003 and *t*(27) = 3.162, *p* = 0.004, respectively (see Figure 3). A one-way ANOVA revealed that these differences did not differ significantly between groups, *F* < 1. These results indicate conscious knowledge by the zero-correlation criterion in both age groups.

**FIGURE 4:**
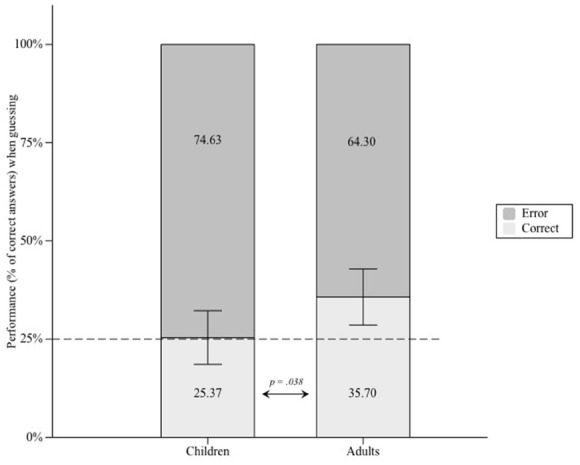
**Proportion of correct and incorrect completions when participants who performed above chance level in the completion task claimed to guess, represented separately for children and adults.** Error bars represent 95% of confidence intervals around the means.

Participants who performed above chance in the completion task did so even when they claimed to guess the correct shape [in 30.839% of the cases, SD = 17.834%, *t*(50) = 2.338, *p* = 0.023]. Nevertheless, a one-way ANOVA indicated that these scores differed as a function of Group, *F*(1,50) = 4.571, *p* = 0.038 (see Figure 4), revealing that this was only the case for young adults who performed above chance [35.702%, SD = 18.108%, *t*(26) = 3.071, *p* = 0.005], but not for children (25.368%, SD = 16.178%, *t* < 1). According to the guessing criterion, these results suggest that completion performance was at least partly based on unconscious knowledge in young adults, but not in children. Besides, when they reported to remember the correct shape, children and adults’ performances did not significantly differ from each other (41.904%, SD = 13.266 and 50.227%, SD = 22.796, respectively), *F*(1,50) = 2.456, *p* = 0.124.

Nevertheless, to ascertain that this null result denotes an absence of implicit knowledge in the children group (null hypothesis), we computed a Bayes factor (BF), the value of which indicates evidence either for the null or for the tested hypothesis. Values below 1/3 constitute evidence for the null hypothesis; values above 3 support the experimental hypothesis. A BF of 1 is exactly neutral between the two theories, and values between 1/3 and 3 reflect insensitive data on the basis of which no strong conclusions can be drawn. We computed the BF on the difference between children performance when they report to guess and a chance level of 25%. Mean difference was 0.368%, with a standard error of 3.315 (derived from the *t*-value of the one-sample *t*-test against chance, 0.111). We used a normal distribution as to remain neutral regarding the direction of the difference between children and adults. This distribution has a mean of 10.702% (i.e., the mean difference between performance and chance level in the adult group, also when they claim to guess) and a standard deviation of 5.351%. In other words, we ran that Bayes analysis in order to make sure that the difference observed between chance level and completion scores in children does not belong to the distribution of the corresponding difference in adults. Using an online calculator^3^, we obtained a BF of 0.14 (<1/3), indicating evidence for the null hypothesis^4^.

### DID COMPLETION PERFORMANCE REVEAL ALL THE ACQUIRED KNOWLEDGE?

We investigated whether participants who performed at chance in the completion task (*n* = 45) actually learned the triplets during exposure based on their results in the indirect RSVP task.

A mixed measures ANOVA with Group as a between-subjects factor and Position as a within-subject factor revealed that RTs differed between target positions in the RSVP task, *F*(2,86) = 15.343, *p* < 0.001, = 0.263. RTs were faster in Position 3 than in Positions 1 and 2 (429 vs. 467 and 472 ms, respectively), both *p* < 0.001.^5^ As in the overall analysis, the main effect of Group was significant, *F*(1,43) = 71.473, *p* < 0.001, ${\text{η}}_{\text{p}}^{\text{2}}$ = 0.624, and indicates that young adults (407 ms, SD = 38) responded faster than children (504 ms, SD = 66 ms). The interaction between both factors was not significant, *F*(2,86) = 1.573, *p* = 0.213.

The observed dissociation between direct and indirect measures of learning in participants who performed at chance in the completion task supports the idea that, in both age groups, these participants acquired statistical knowledge that cannot be used intentionally in the direct task.

## DISCUSSION

In this study, 9- to 12-year-old children and young adults took part in a VSL experiment in which learning was assessed indirectly through a RSVP detection task and directly through a 4AFC completion task. Subjective confidence judgments were also collected in this latter task in order to investigate the explicit or implicit nature of the acquired knowledge.

Rapid serial visual presentation results show that participants from both age groups learned in a similar fashion. The results of the completion task indicate that about half the participants were able to use their knowledge to identify the missing shape in incomplete triplets. Results also show that those who performed at chance in the completion task nevertheless learned the regularities as assessed by their RT results.

Children and adults’ completion performances do not differ from each other. Our results therefore suggest that VSL abilities are mature at 10 years of age. Nevertheless, as we will discuss below, the nature of their knowledge may not be identical.

At first sight, this may seem surprising, as previous studies have reported age effects in spatial and temporal SL. Indeed, even though most authors investigating the developmental aspects of SL have observed successful learning both in adults and children groups (Dixon et al., 2010; Couperus et al., 2011; Arciuli and Simpson, 2012; but see Vaidya et al., 2007), more effective SL has usually been observed in adults than in children (but see Saffran et al., 1997). Investigating VSL in 5–12 years, Arciuli and Simpson (2011) reported that learning improves with age, also supporting that SL follows a developmental trajectory.

The fact that children and adults performance did not differ from each other does not necessarily speak against these conclusions. Most probably, the difficulty of the completion task may have weakened potential differences in performance between age groups (even in adults, the average performance reached only 32%, while chance level was 25%). Using a wider range of children’s ages would presumably induce stronger developmental effects, with children performance increasing with age, as observed by Arciuli and Simpson (2011).

However, as we had to measure confidence to investigate age differences in the nature of the acquired knowledge, we did not recruit younger children. Indeed, children under 9 years of age may be less able to assess the correctness of their responses (Koriat and Ackerman, 2010). Further studies using the same paradigm (although without or with adapted confidence judgments) should investigate the developmental course of implicit and explicit VSL across younger children.

Although average performance was above chance, approximately half of the participants in each group responded at chance in the completion task. Interestingly, those participants showed intact learning in the RSVP task. This result supports that these participants acquired knowledge of the sequences that cannot be used intentionally in the completion task. Such dissociation between direct and indirect performance has often been considered as an indication of implicit learning (e.g., Kim et al., 2009). However, as convincingly shown by Shanks and Perruchet (2002), such dissociation between tasks does not necessarily indicate a corresponding dissociation between conscious and unconscious knowledge. Rather, it may merely reflect the fact that both measures are imperfectly correlated and not equally sensitive to the acquired knowledge. As a consequence, the simple dissociation between direct and indirect performance provides only moderate evidence for implicit VSL in both age groups. More interesting is the qualitative dissociation between performance and confidence in participants who performed above chance in the completion task.

The other half of participants in each group reached above chance performance in the completion task. Looking at the subjective confidence measures, we checked the amount of correct completions made with no confidence. In young adults, completion scores were above chance even when they claimed to guess the missing shape. Such an effect has been previously reported in the implicit learning literature. For example, in artificial grammar learning studies where participants had to classify strings of letters as grammatical or not, several authors (Dienes et al., 1995; Tunney and Shanks, 2003; Scott and Dienes, 2008) have found above chance performance even when participants attributed responses to random selection. Other studies, using the sequence learning paradigm have also found that participants cannot help generating the training sequence above the chance level when instructed not to do so (Destrebecqz and Cleeremans, 2001; Fu et al., 2008). Such a result suggests that the expression of the acquired knowledge was not entirely under conscious control. According to Scott and Dienes (2008), these effects are consistent with the notion that behavior reflects, at least to some extent, the unconscious influence of familiarity.

Further results in the adult group showed that accuracy in the completion task was reliably related to confidence however. In particular, these adults who performed above chance were more confident when they correctly identified the missing shape than when they were mistaken. Hence, the pattern of results found in adults corresponds to the typical observation that subjective measures indicate the existence of some unconscious knowledge according to the guessing criterion and some conscious knowledge according to the zero-correlation criterion. Typically, participants acquire both conscious and unconscious knowledge (Dienes and Scott, 2005; Bertels et al., 2012, 2013).

Surprisingly, children’s performance does not show unconscious influence on performance, neither through the guessing criterion nor through the zero-correlation criterion, although adults’ does (at least through the guessing criterion). This is in contradiction with Daltrozzo and Conway’s (2014) model that postulates a greater involvement of implicit mechanisms (i.e., of the “basic” system) in childhood than in adulthood. On the basis of this model, we indeed expected that children’s performance would rely more on implicit and less on explicit knowledge than adults’ performance. Specifically, we predicted that (1) children would outperform adult when they both reported to guess and that (2) the difference in confidence level reported in correct and incorrect trials would be larger in adults than in children. We instead observed that (1) children, unlike adults, performed at chance when they claimed to guess and that (2) a similar difference in confidence between correct and incorrect trials was found in both age groups.

Should we then consider learning in the children group as more “conscious” than in the adult group? Not necessarily. In his theoretical model of unconscious cognition, Cleeremans (2006) proposed that availability to consciousness may paradoxically decrease with the acquisition of “high quality” representations. In his framework, “quality of representation” designates several properties of memory traces, such as their relative strength, their distinctiveness, or their stability in time. The model also states that the emergence of high-quality representations takes time over development. At first, learning is best described as “implicit” because the relevant representations are too weak to be controlled by the system. At a second stage, explicit representations begin to emerge and may serve as the basis of responding in direct measures of learning. Finally, as the quality of the representations continues to increase through practice or over development, the corresponding content of phenomenal experience may simultaneously decrease as these representations are no longer part of the central focus of awareness and as their potential influence on behavior does not need to be consciously monitored anymore. According to Cleeremans (2008), “conscious experience occurs if and only if an information processing system has learned about its own representations of the world” (p. 23). In his view, contents can be accessed consciously “provided […] that the system has learned about its representations by itself, over its development” (p. 23). Hence, in our study, adults may require less focus on the relevant regularities to reach a similar level of performance as children. This may explain why children performance revealed less unconscious influences than adults when subjective measures are used.

Arguably, our results may be biased by the children’s high confidence (at least higher than the level of confidence found in adults). For instance, if children expressed too much confidence in their errors, one might have expected a smaller Chan difference in children than in adults. As Chan differences are computed on confidence judgments considered separately for correct and erroneous responses, such a difference would reflect a bias in children’s assessment of confidence rather than a true difference in the nature of learning taking place in both age groups^6^. In particular, one might have predicted the Chan difference to be smaller in children than in adults if children were specifically too confident in their errors. However, Chan differences were similar and reliably above 0 in both age groups.

A liberal metacognitive bias in children may have also resulted in lower performance in “remember” trials in children than in adults. Indeed, being overconfident would lead children to report to remember the answer although it was incorrect. However, children and adult performance did not statistically differ from each other. Taken together, these data support that the children higher level of confidence did not influence completion performance and the criteria we used to assess the nature of their statistical knowledge.

Why were children more confident than young adults, although the same instructions were given in both groups? We cannot rule out the possibility that the presence of an adult experimenter who accompanied children through the task urged them to report confidence, either because the children did not want to admit they were unconfident in front of that person or because she was so reinsuring that she boosted children’s self-confidence. This point should be controlled for in further studies. It is also possible that children and adults understood the instructions in different ways. However, as described in the next section, we tried to make the instructions as clear and explicit as possible in order to avoid any misunderstanding in participants in both age groups.

The two labels we used for the confidence judgments were chosen in order to obtain the most exhaustive measure of conscious knowledge possible. As we did in our previous studies, we made clear to the participants that they had to say “Guess” when they had no idea or intuition whatsoever concerning the missing shape, they could just as well flip a coin to determine their answer. We explicitly told them that a “Guess” meant no conscious feeling of familiarity or recall whatsoever. This was done in order to ensure that above chance performance when claiming to guess effectively measures the influence of implicit knowledge. Alternatively, they had to say “Remember” following any recall of the learning material, even held with very low confidence. Still, in case participants’ response was based on intuition, one might wonder which option to choose. Indeed, although a response based on intuitive feelings implies that the participant is not completely guessing, he/she did not recall the learning material either. Therefore, further studies should use clarified labels such as “Guess” vs. “Some confidence,” without referring to any recall of the material. Intuitive responses would then clearly be associated to the “Some confidence” option.

An additional scale should be used to obtain a more precise assessment of the knowledge held by participants with some level of confidence. Indeed, while our subjective measures are concerned with what Dienes and Scott (2005) have called “judgment knowledge,” i.e., knowing of *what* is the missing shape, we have no information about what they have called “structural knowledge,” i.e., knowing of *why* this is the missing shape. In some cases, adults judged that they were guessing while they were above chance. Such a pattern of results indicates unconscious judgment knowledge, which is necessarily associated with unconscious structural knowledge. If one does not know *that* he knows the missing shape, he could not know *why* he knows it. In the children group by contrast, participants demonstrated conscious judgment knowledge. But conscious judgment knowledge can be coupled with either conscious or unconscious structural knowledge. Children may know that they know which shape is the missing shape but their corresponding phenomenal experience may be intuition rather than conscious identification of the reasons why a given shape is the missing one (see Dienes and Scott, 2005, p. 339). Therefore, in further studies, we recommend (1) to assess judgment knowledge by using binary confidence judgments with clarified labels such as “Guess” vs. “Some confidence,” and (2) to ask participants to indicate after each judgment the basis of their judgment (e.g., guess, intuition, rules, memory, as in Dienes and Scott, 2005). This will be useful in testing whether children outperform adult participants regarding the conscious status of their structural knowledge.

### Conflict of Interest Statement

The authors declare that the research was conducted in the absence of any commercial or financial relationships that could be construed as a potential conflict of interest.

## Footnotes

1 Another ANOVA was performed on z-scores of RT in order to cancel any effect of the difference in response speed between groups. This analysis confirmed the significant main effect of Position. The interaction between Position and Group and the main effect of Group turned out to be non-significant (both *F* < 1).

2 This analysis was chosen because completion scores were not normally distributed. An additional one-way ANOVA on normalized data (square root transformation) also indicated that the main effect of Group was non-significant [*F*(1,96) = 1.472, *p* = 0.228].

3 http://www.lifesci.sussex.ac.uk/home/Zoltan_Dienes/inference/Bayes.htm

4 However, one could argue that, since we observed higher performance in adults than in children when they reported to guess, we should use a uniform rather than a normal distribution when computing the Bayes factor. Using an uniform distribution ranging from 0 to 10.702, we obtained a BF of 0.42, indicating more support for the null hypothesis than for the existence of an effect (as it is smaller than 1), but also indicating that the data are not sensitive enough to firmly conclude in one or the other direction. More data should be collected in further studies.

5 Interestingly, a mixed measures ANOVA including the between-subjects factor Chance Level in the completion task (two levels: Above/At) revealed that this effect of Position was not modulated by Chance Level (*F* < 1), suggesting that both groups of participants performed similarly in the RSVP detection task.

6 We are aware that most authors (including Bertels et al., 2012, 2013) submitted confidence judgments to Type II SDT analysis, in which Hits correspond to correct discriminations made with high confidence and False Alarms to incorrect discriminations made with high confidence (Kunimoto et al., 2001). This procedure results in Type II *d*′ representing participants’ awareness of their own performance and claimed to be an unbiased measure of awareness, unaffected by participants’ own report criterion in making high and low confidence judgments (Kunimoto et al., 2001; Tunney and Shanks, 2003). Nevertheless, recent studies have shown that Type II *d*′ are in fact not independent from metacognitive bias (e.g., Galvinet et al., 2003; Evans et al., 2007; Barrett et al., 2013; Fleming and Lau, 2014). We therefore did not use this analysis in the present study. We thank an anonymous reviewer for bringing this literature to our attention.
